# Do We Reap What We Sow? Exploring the Association between the Strength of European Primary Healthcare Systems and Inequity in Unmet Need

**DOI:** 10.1371/journal.pone.0169274

**Published:** 2017-01-03

**Authors:** Jens Detollenaere, Lise Hanssens, Veerle Vyncke, Jan De Maeseneer, Sara Willems

**Affiliations:** Department of Family Medicine and Primary Health Care, Ghent University, Ghent, Belgium; UNAIDS, GUYANA

## Abstract

Access to healthcare is inequitably distributed across different socioeconomic groups. Several vulnerable groups experience barriers in accessing healthcare, compared to their more wealthier counterparts. In response to this, many countries use resources to strengthen their primary care (PC) system, because in many European countries PC is the first entry-point to the healthcare system and plays a central role in the coordination of patients through the healthcare system. However it is unclear whether this strengthening of PC leads to less inequity in access to the whole healthcare system. This study investigates the association between strength indicators of PC and inequity in unmet need by merging data from the European Union Statistics on Income and Living Conditions database (2013) and the Primary Healthcare Activity Monitor for Europe (2010). The analyses reveal a significant association between the Gini coefficient for income inequality and inequity in unmet need. When the Gini coefficient of a country is one SD higher, the social inequity in unmet need in that particular country will be 4.960 higher. Furthermore, the accessibility and the workforce development of a country’s PC system is inverse associated with the social inequity of unmet need. More specifically, when the access- and workforce development indicator of a country PC system are one standard deviation higher, the inequity in unmet healthcare needs are respectively 2.200 and 4.951 lower. Therefore, policymakers should focus on reducing income inequality to tackle inequity in access, and strengthen PC (by increasing accessibility and better-developing its workforce) as this can influence inequity in unmet need.

## Introduction

The socioeconomic conditions in which people live play a large part in influencing their chances of living a healthy life [1–4]. Access to healthcare is an important and fundamental indicator of health, and its equitable distribution across patients is a never-ending concern within health services research [5–8]. Equitable access to care should be determined by a patient’s need for medical care and not by their social status, age, gender, income or ethnic background [9]. In the present time however, people from some social groups experience more barriers in accessing primary care (PC) compared with other social groups [10–16]. In response to this inequitable distribution of access, many countries aim to improve access to healthcare by strengthening their PC systems. However, until now, it remains unclear whether strong PC systems are associated with equity in access to healthcare.

For those in need, access to healthcare has a positive influence on self-perceived health and life expectancy [17, 18]. Moreover, good health outcomes at a national level are related to beneficial economic outcomes, such as productivity and output [17]. Therefore, it is not surprising that providing citizens with adequate access to healthcare services has been a major goal of many European policymakers. According to several European policy documents (e.g., EU Charter for Fundamental Rights, Treaty on the Functioning of the EU and the International Covenant on Economic, Social, and Cultural Rights) it is the responsibility of all European Union (EU) member states to establish a right of access to core healthcare services for everyone, especially vulnerable and marginalised patients, with an equitable distribution based on health needs [17]. However, notwithstanding the intentions of these policy documents, there is still great variation among the proportions of populations reporting unmet healthcare needs across Europe. The organisation and financing of PC in European countries is characterised by a variety of delivery models, but, recent reforms have led to an increase in convergence [19]. Various disciplines are involved in PC delivery, although GPs in Europe are usually the main PC actors and guide patients through the healthcare system [20]. These GPs are almost always self-employed, and paid through a mix of fee-for-service and capitation payment systems. Additionally, most European countries use the GP as gatekeeper and financial incentives to regulate access to secondary care [19].

The most commonly used measure of access to healthcare is self-assessed unmet need [21]. Carr and Wolfe [22] define unmet healthcare needs as ‘the differences, if any, between those services judged necessary to deal appropriately with defined health problems and those services actually being received […] an unmet need is the absence of any, or of sufficient, or of appropriate care and services’. This definition is the most suitable method for measuring unmet healthcare need. This subjective assessment of unmet healthcare need perceives the patient to be the best assessor of their health status and whether they have received the most convenient healthcare [23].

Reported unmet need ranges from less than 1% in Slovenia and Belgium to 26% in Latvia [24]. In addition, the prevalence of unmet healthcare need appears to be increasing over time. From 2005–2008 unmet healthcare need in the EU decreased by 2%; however this downward trend reversed from 2008–2013, when the prevalence of unmet need began to grow again. It reached 3.6% in 2013. According to Reeves, McKee [25], more than 1.5 million additional people have reported unmet healthcare needs since the beginning of the financial and economic crisis. This reversing trend can be explained by the onset of the financial and economic crisis and the related introduction of austerity measures in several European countries [17, 26], especially in countries with a large income inequality [26]. Reeves, McKee [25] identified demand-side factors (e.g., increasing co-payments, rising transport costs and reduced incomes) and supply-side factors (e.g., closing times of health facilities and reduction in opening hours) as potential mechanisms underlying this evolution. Furthermore, a recent European contribution shows that countries with a large income inequality were associated with a higher prevalence in unmet need. This effect occurred only among the disadvantaged population in a European country, and among the more wealthier population groups. The scarce literature available identifies low income as one of the strongest predictors of experiencing unmet need [21, 27, 28]. Receiving an adequate income is essential to being able to purchase healthcare and is vital for obtaining access to PC and specialist care.

As mentioned above, this article intends to explore whether the strength of European PC is associated with inequality in unmet need. To the best of our knowledge, the present study is the first attempt to address this association with an international comparison. Nonetheless, the existence of this association is supported by previous studies that have provided evidence of the positive influence of PC strength on several other health(care)-related measures. For example, strong PC is associated with better population health [29–31], improved quality of care [32], reduced socioeconomic inequality in health [29], higher self-rated health for people with chronic diseases [33] and better cost control [34]. The positive influence of PC strength on health outcomes can be attributed to the main characteristics of PC: providing accessible, comprehensive care in an ambulatory setting to patients in their own context on a continuous basis and coordinating the care processes of patients across the healthcare system [35]. Moreover, PC can act as a mediator for relatively deprived population groups, and in doing so may increase accessibility to other healthcare services [36]. PC functions as the first point of contact with a healthcare system and facilitates entry to the rest of the system.

Besides the fact that previous literature on unmet healthcare need has not addressed the link between the strength of PC and socioeconomic inequalities in unmet need, it is also characterised by other limitations. Firstly, most of the existing literature on unmet need comprises single-country studies (conducted mainly in the US and Canada). In addition, few of these studies are based on general population groups [28, 37], while most focus on specific patient groups [27, 38–42], thereby limiting the generalisability of their findings. Moreover, only a limited number of studies with international comparisons have been conducted [6, 27, 43, 44]. Finally, most previous studies in this field focus on the prevalence of, rather than the inequity in, unmet need, while policymakers are particularly interested in the latter aspect. An exception to this is a recent study by Chaupain-Guillot and Guillot [27] which investigated the relationship between health system characteristics and unmet need across European countries.

In the present study we build on and contribute to the mentioned body of literature by answering the following research question: is the strength of European PC systems associated with income-driven inequity in unmet healthcare need at the macro level? In other, more poetic words, do we reap social inequity in unmet need, when sowing weak PC systems?

## Materials and Methods

To answer the research question, data from two European databases were combined: (i) data on national unmet healthcare needs from the 2013 wave of European Union Statistics on Income and Living Conditions (EU-SILC) and (ii) data on the strength of the national PC systems from the Primary Healthcare Activity Monitor for Europe (PHAMEU) (2010).

### Data and operationalisation

The EU-SILC, gathered under the coordination of Eurostat, is the EU reference source for comparative statistics on income distribution and social inclusion at the European level [45]. EU-SILC provides two types of data concerning the 27 EU countries, as well as Croatia, Iceland, Norway, Switzerland, and Turkey: (i) longitudinal data containing individual-level changes over time, observed periodically over four years and (ii) cross-sectional data on income, poverty, social exclusion and living conditions. The minimum size of the surveyed population each year is approximately 100,000 households and 200,000 citizens aged 16 years or over for the longitudinal part of the study, and 130,000 households and 270,000 citizens aged 16 years or over for the cross-sectional data. The 2013 wave of data (used for the current study) included the 27 Member States of the European Union, as well as Norway and Iceland. However EU-SILC did not provide data on unmet healthcare needs for some countries for 2013. For these countries, the authors used the data from the most recent wave provided in EU-SILC (for Malta, the Netherlands, Austria and FYR Macedonia this was 2012, for Sweden this was 2009, for Norway this was 2008, for Turkey this was 2007 and for Slovenia this was 2005).

Access to healthcare was measured by asking participants: ‘Was there any time during the last 12 months when, in your opinion, you needed medical examination or treatment […] but you did not receive it?’ If participants answered ‘yes’ to this question, they were categorised as participants who suffered from unmet healthcare need. Inequity (or the gap) in unmet healthcare need was calculated by subtracting the percentage of participants in the lowest quintile of equivalised income reporting unmet need by the percentage of participants in the highest quintile of equivalised income reporting unmet need. Equivalised income is the total income of a household, after tax and other deductions, divided by the number of household members. To convert the household members into equalised adults, they were each weighted according to their age using the modified OECD equivalence scale [46]. This approach to calculating inequity through the interquintile range is similar to that used in previous studies [47, 48]. Kalmijn and Veenhoven [48] explored several statistics to operationalise income-driven inequity across countries. Compared to other statistics (for instance the mean absolute difference), they concluded that the interquartile range proved suitable for operationalising inequity. In the present study, due to the fact that publicly available data was only provided by means of quintiles, the authors were forced to base their measure on interquintile instead of interquartile ranges.

Secondly, given the complexity of and variation in European PC, PHAMEU was used to determine the strength of the national PC systems, and by doing so, made the complex European PC landscape comparable. The seven strength indicators of PHAMEU capture a combination of PC functions both at the structure level (governance, economic conditions and workforce development) and at the process level (access, continuity, coordination and comprehensiveness) [49]. A detailed overview of the specific composition of these strength indicators is provided in *Table 1*.

**Table 1:** Framework of the European Primary Care Monitor.

	Description by Kringos (2012)	Components
**Strength of PC structure**		
Governance	Oversees all aspects of PC. It encompasses the tasks of defining the vision and direction of health (care) policy, exerting influence through regulation and advocacy, and collecting and using information.	1. PC goals 2. Policy on equality in access to PC 3. (De)centralization of PC management and service development 4. Quality management infrastructure 5. Patient advocacy 6. Multidisciplinary collaboration
Economic conditions	Are to a great extent shaped by the method of financing healthcare for the population, total expenditures on healthcare and PC, etc.	1. PC expenditure 2. PC coverage 3. Remuneration system of PC workforce 4. Income of PC workforce
Workforce development	Shaped by the profile of PC professionals that make up the PC workforce in a country, and the position they occupy in the healthcare system.	1. Profile of PC workforce 2. Status and responsibilities of PC disciplines 3. PC workforce supply and planning 4. Academic status of PC 5. Medical associations
**Strength of PC process services delivery**		
Access	Can be defined as the ease with which PC services are reached by patients.	1. Density PC workforce 2. Geographic availability of PC services 3. Accommodation of accessibility 4. Affordability of PC services 5. Acceptability of PC services
Continuity	Conditions related to enduring doctor-patient relationships.	1. Longitudinal continuity of care 2. Information continuity of care 3. Relation continuity of care
Coordination	The ability of PC providers to guide the use of care with other levels of healthcare or other healthcare providers, so that providers can work together to meet patients’ needs.	1. Gatekeeping system 2. Skill-mix of PC providers 3. Collaboration of PC-secondary care 4. Integration of public health in PC
Comprehensiveness	Describes the extent to which PC provides the most comprehensive scope of health services within a healthcare system and address the wide variety and often very basic needs existing in the community.	1. Medical equipment available 2. First contact for common health problems 3. Treatment and follow-up of diseases 4. Medical technical procedures 5. Preventive care 6. Mother and child & reproductive healthcare 7. Health promotion

For additional information about the selection of the indicators, data collection, and calculation of the scales see Kringos [35]. These European Primary Care Monitor components were used to calculate seven separate scores (one for each indicator of strength) via a two-level hierarchical regression model. For all countries, the scores for these seven strength dimensions are listed in *table 1* as percentiles (33 and 67) rather than the actual five digit decimals to facilitate interpretation.

### Statistical analysis

The data were analysed using SPSS (version 23.0.0, IBM). The distribution of the dependent variable (social inequity in unmet healthcare need) and the governance-indicator was highly skewed, and because they were rejected by the normal distribution hypothesis using the Shapiro-Wilk test, these two variables were logarithmic transformed.

Firstly, the dependence between the seven aforementioned strength indicators (each time used as a scale) and the gap in unmet healthcare need between low- and high-income groups was tested using Pearson correlation coefficients. Secondly, multiple linear regression models were used to assess the relative and independent contribution of the seven strength indicators to the gap between low- and high-income groups in unmet healthcare need. In the second regression model, we additionally controlled for the unequal distribution of countries’ wealth by adding the Gini index of income inequality to the model. The World Bank [50] defines the Gini index of income inequality as the extent to which the distribution of income among individuals or households within an economy deviates from a perfectly equal distribution. No variables required exclusion due to multicollinearity issues. The level of statistical significance was set at p ≤ 0.05.

## Results

Firstly, we provide the reader with a brief summary of European PC strength (*Table 2*). According to PHAMEU, the countries that scored the highest (lowest) on the governance-indicator were the Netherlands and Spain (Switzerland and Luxembourg). Furthermore, concerning economic conditions, the United Kingdom and Spain (Bulgaria and Ireland) scored the highest (lowest). The United Kingdom and the Netherlands (Iceland and Luxembourg) had the best (weakest) developed workforce.

**Table 2:** Overview of the country characteristics in relation to healthcare system features: structure and process strength.

Country	Strength PC Structure	Governance	Economic conditions	Workforce development	Strength PC process	Access	Continuity	Coordination	Comprehensiveness
Austria	Weak	Medium	Medium	Weak	Weak	Medium	Weak	Weak	Medium
Belgium	Medium	Medium	Strong	Medium	Medium	Weak	Strong	Medium	Strong
Bulgaria	Weak	Medium	Weak	Weak	Medium	Weak	Medium	Weak	Strong
Cyprus	Weak	Weak	Weak	Weak	Weak	Weak	Medium	Weak	Weak
Czech Republic	Weak	Medium	Weak	Weak	Medium	Strong	Strong	Medium	Weak
Denmark	Strong	Strong	Medium	Strong	Strong	Strong	Strong	Strong	Medium
England	Strong	Strong	Strong	Strong	Strong	Strong	Medium	Strong	Strong
Estonia	Medium	Strong	Weak	Medium	Strong	Medium	Strong	Medium	Medium
Finland	Strong	Weak	Strong	Strong	Strong	Medium	Weak	Medium	Strong
FYR Macedonia	-	-	-	-	Weak	Strong	Weak	Weak	Weak
Germany	Medium	Medium	Strong	Weak	Weak	Medium	Strong	Weak	Medium
Greece	Weak	Medium	Weak	Weak	Weak	Weak	Weak	Strong	Weak
Hungary	Weak	Weak	Medium	Medium	Medium	Strong	Medium	Weak	Medium
Iceland	Weak	Weak	Weak	Weak	Medium	Medium	Strong	Medium	Medium
Ireland	Medium	Weak	Weak	Strong	Weak	Weak	Strong	Weak	Weak
Italy	Strong	Strong	Strong	Medium	Medium	Medium	Medium	Medium	Weak
Latvia	Weak	Medium	Medium	Weak	Medium	Weak	Medium	Medium	Medium
Lithuania	Medium	Medium	Medium	Medium	Strong	Medium	Weak	Strong	Strong
Luxembourg	Weak	Weak	Weak	Weak	Weak	Weak	Weak	Medium	Medium
Malta	Medium	Weak	Weak	Strong	Medium	Medium	Weak	Strong	Strong
Netherlands	Strong	Strong	Strong	Strong	Strong	Strong	Weak	Strong	Weak
Norway	Medium	Strong	Weak	Medium	Medium	Medium	Medium	Weak	Strong
Poland	Weak	Weak	Weak	Weak	Strong	Strong	Medium	Strong	Weak
Portugal	Strong	Strong	Medium	Strong	Strong	Strong	Medium	Medium	Strong
Romania	Medium	Strong	Medium	Medium	Weak	Medium	Medium	Weak	Weak
Slovakia	Weak	Weak	Medium	Weak	Weak	Medium	Strong	Weak	Weak
Slovenia	Strong	Strong	Strong	Strong	Strong	Strong	Weak	Strong	Weak
Spain	Strong	Strong	Strong	Strong	Strong	Strong	Strong	Strong	Strong
Sweden	Medium	Medium	Medium	Medium	Strong	Medium	Weak	Strong	Strong
Switzerland	Weak	Weak	Medium	Medium	Medium	Medium	Medium	Medium	Strong
Turkey	Medium	Medium	Strong	Medium	Weak	Weak	Weak	Weak	Medium

**Source:** authors’ calculations based on PHAMEU (2010)

Moreover, the highest (lowest) accessibility was reported in Slovenia and Denmark (Ireland and Luxembourg). Regarding continuity, Denmark and Estonia (Turkey and Malta) were the strongest (weakest). Sweden and the Netherlands (Austria and Germany) had the strongest (lowest) PC coordination. Countries that provided the best (weakest) comprehensive care were Lithuania and Bulgaria (FYR Macedonia and Slovakia). In short, although other countries often had the strongest (weakest) scores on several strength-indicators, the Netherlands, Spain and the UK primarily showed to be the strongest concerning PC, while Luxembourg scored the weakest in this respect.

**Fig 1:**
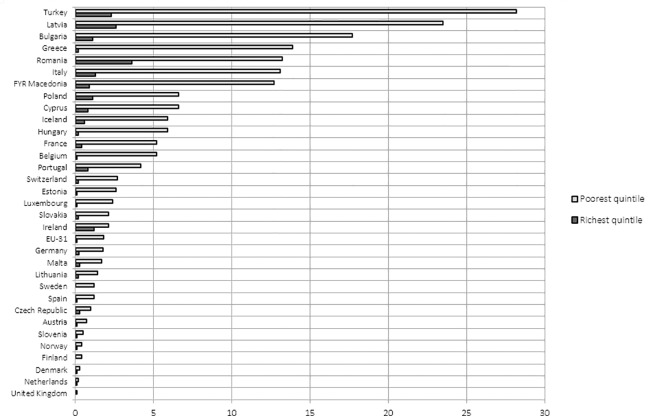
Percentage of people reporting unmet health care needs, comparing the highest and lowest income quintile. **Source** Authors’ representation based on the EU-SILC data (2013)

**Fig 2:**
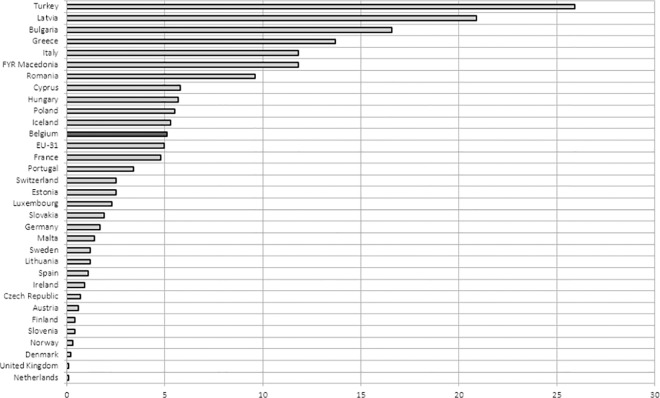
Gap unmet health care needs between low and high income groups. **Source** Authors’ representation based on the EU-SILC data (2013)

*Fig 1* shows how unmet healthcare need differed by income level. In all European countries, people in the lowest income group reported the highest unmet need. The highest percentage of the population reporting unmet need was observed in Turkey (28.2%) and the lowest in the United Kingdom (0.1%). The country with the highest social inequity in unmet healthcare need between low- and high-income groups was Turkey. Consequently, Turkey reported the highest inequity in unmet healthcare need. The Netherlands had the lowest gap and therefore reported the lowest inequity in unmet healthcare need *(Fig 2)*.

To present the univariate association between the social gap in unmet healthcare need and the seven strength indicators of the PC system, Pearson correlation coefficients were calculated. *Table 3* presents the results of this correlation matrix. We observed a significant correlation between unmet healthcare need and economic condition (R: -0.384, p 0.036), workforce development (R: -0.551, p 0.002), access (R: -0.451, p 0.011) and coordination (R: -0.380, p 0.035). Each of these correlations showed that the higher the score on the indicator, the lower the gap in unmet need. Furthermore, the matrix revealed a significant correlation between the Gini coefficient for income inequality and the gap in unmet healthcare need (R: 0.421, p 0.017). There were no associations between the gap in unmet healthcare need and governance, continuity and comprehensiveness.

**Table 3:** Correlation matrix between the dependent variable and all independent variables.

	Gap unmet need	GINI index for income inequality	Governance	Economic conditions	Workforce development	Access	Continuity	Coordination	Comprehensiveness
**Gap unmet need**		**0.355 (0.046)**	- 0.236 (0.209)	**- 0.384 (0.036)**	**- 0.551 (0.002)**	**- 0.451 (0.011)**	- 0.152 (0.413)	**- 0.380 (0.035)**	- 0.278 (0.130)
**GINI index for income inequality**	**0.355 (0.046)**		0.127 (0.502)	0.195 (0.302)	0.264 (0.159)	- 0.062 (0.741)	- 0.114 (0.540)	0.012 (0.947)	- 0.227 (0.220)
**Governance**	- 0.236 (0.209)	0.127 (0.502)		**0.372 (0.043)**	**0.383 (0.037)**	**0.495 (0.005)**	- 0.031 (0.872)	**0.385 (0.036)**	0.184 (0.330)
**Economic conditions**	**- 0.384 (0.036)**	0.195 (0.302)	**0.372 (0.043)**		**0.488 (0.006)**	**0.478 (0.008)**	- 0.036 (0.850)	0.268 (0.152)	0.081 (0.669)
**Workforce development**	**- 0.551 (0.002)**	0.264 (0.159)	**0.383 (0.037)**	**0.488 (0.006)**		0.313 (0.092)	- 0.040 (0.833)	**0.380 (0.038)**	0.301 (0.106)
**Access**	**- 0.451 (0.011)**	- 0.062 (0.741)	**0.495 (0.005)**	**0.478 (0.008)**	0.313 (0.092)		0.249 (0.177)	0.234 (0.205)	- 0.057 (0.761)
**Continuity**	- 0.152 (0.413)	- 0.114 (0.540)	- 0.031 (0.872)	- 0.036 (0.850)	- 0.040 (0.833)	0.249 (0.177)		- 0.194 (0.295)	0.146 (0.434)
**Coordination**	**- 0.380 (0.035)**	0.012 (0.947)	**0.385 (0.036)**	0.268 (0.152)	**0.380 (0.038)**	0.234 (0.205)	- 0.194 (0.295)		0.245 (0.183)
**Comprehensiveness**	- 0.278 (0.130)	- 0.227 (0.220)	0.184 (0.330)	0.081 (0.669)	0.301 (0.106)	- 0.057 (0.761)	0.146 (0.434)	0.245 (0.183)	

All significant results are indicated in bold

To determine the independent impact of the strength indicators on the observed gap in unmet healthcare need, an initial multiple linear regression model was estimated *(Table 4)*. This model showed significant associations between two of the seven strength indicators and explained 29.5% of the variance in inequity in unmet healthcare need. Consistent with the correlation matrix, the association between access and unmet healthcare need remained significant in the regression model. Access was inverse associated with the gap in unmet healthcare need (p 0.020). The better the access to the PC system, the smaller the gap in unmet healthcare need within a country. More specifically, when the access-indicator is one standard deviation higher, the inequity in unmet need is about 4.371 lower. Secondly, we observed an inverse association between workforce development and the gap in unmet healthcare need (p 0.047). In other words, the better developed the PC workforce is, the lower the inequity in unmet healthcare need. Specifically, when the workforce development of a country is one standard deviation higher, the inequity in unmet need of this particular country is 3.967 lower. The significant correlation in the bivariate analysis for economic conditions and coordination disappears in the multiple regression model. Furthermore, the other three strength indicators (governance, continuity and comprehensiveness) had no significant impact on the gap in unmet healthcare need.

**Table 4:** Linear regression of the gap between low and high income groups on PC strength indicators, and in the second linear regression controlling for the GINI index for income inequality.

	Model 1			Model 2		
	B	SD	p	B	SD	P
**Constant**	22.784	11.802	0.067	21.241	10.340	0.053
**GINI index for income inequality**				**4.960**	**0.057**	**0.011**
**Strength PC structure**						
Governance	5.534	5.485	0.324	3.436	4.858	0.487
Economic conditions	- 0.348	2.987	0.908	- 1.789	2.664	0.509
Workforce development	**- 3.967**	**1.888**	**0.047**	**- 4.951**	**1.689**	**0.008**
**Strength PC process**						
Access	**- 4.371**	**2.393**	**0.020**	**- 2.200**	**2.234**	**0.018**
Continuity	- 1.948	4.575	0.674	- 2.875	4.017	0.482
Coordination	- 1.143	1.235	0.365	- 1.300	1.082	0.243
Comprehensiveness	- 0.717	1.846	0.701	- 0.246	1.624	0.881
			Adjusted R^2^: 0.295			Adjusted R^2^: 0.460

All significant results are indicated in bold

In the second and final model we controlled for the Gini index for income inequality. This model explained 46.0% of the variance. The association between access and the gap in unmet need on the one hand (p 0.018) and workforce development and the gap in unmet need on the other (p 0.008) remained statistically significant when the Gini index was taken into account. When the access- and workforce development indicator of a country are one standard deviation higher, the inequity in unmet healthcare needs are respectively 2.200 and 4.951 lower. A positive association between the Gini index for income inequality and the gap in unmet healthcare need was shown (p 0.011), indicating that the higher the income inequality, the bigger the gap in unmet healthcare need. Specifically, when the Gini index is one SD higher, the social inequity in unmet need will be 4.960 higher. Finally, the other five strength indicators (governance, economic conditions, continuity, coordination and comprehensiveness) showed no significant associations with the gap in unmet need.

## Discussion

In most European countries some social groups experience barriers in accessing (primary) healthcare and have therefore an inequitable disadvantage compared to their more wealthier counterparts [7, 8]. Many countries use resources to strengthen the PC system and tackle this inequity. However, it is unknown whether strong PC systems are related to less inequity in healthcare accessibility. Therefore, the current study empirically investigated the association between the indicators of the strength of PC and inequity in unmet healthcare need in Europe at the macro level. This study complements recent European contributions which have examined the association between health system characteristics and unmet care need [27] by focusing on (i) the characteristics of the PC system (rather than the total healthcare system) and (ii) the inequity dimension in unmet healthcare need (rather than the prevalence of unmet healthcare need). To that end, we merged data from the 2013 wave of EU-SILC and from PHAMEU (2010).

The results of this study show the largest inequity gap in unmet healthcare need in Turkey. Moreover, according to PHAMEU, Turkey has a weak PC system. Bivariate analyses revealed a significant correlation between the social gap in unmet need and (i) the Gini coefficient for income inequality, (ii) the access-indicator of the strength of PC and (iii) the workforce development-indicator of the strength of PC. Furthermore, according to the estimation results of the multiple linear regression model, two indicators of PC strength predict inequity in unmet healthcare need. Firstly, an inverse effect between access and inequity in unmet healthcare need was observed. In other words, a more accessible primary healthcare system was associated with lower inequity in unmet healthcare need. This is consistent with recent literature, in which unmet healthcare need has been shown to be the most commonly used proxy to measure access to healthcare [21].

Secondly, this study suggests that a better-developed workforce within PC and a more central role of PC professionals (e.g., a gatekeeping role) within the healthcare system is associated with lower inequity in access to healthcare, thus lowering inequity in unmet need [21]. Furthermore, the results of the multiple regression model reveal a significant association between the Gini coefficient for income inequality and the social inequity in unmet need. This result is, to some extent, tautological. Given the fact that social inequity in unmet healthcare need is calculated using income quintiles it is not surprising, and even logical, that there is a significant association between this independent variable and inequity in unmet need. However, this association complements the research of Wilkinson and Pickett [51] that demonstrates the importance of income inequality on health and wellbeing.

Finally, in a recent research, Chaupain-Guillot and Guillot [27] found a positive link between households’ out-of-pocket payments in total health expenditure and the probability of unmet healthcare needs. In this study, we found a significant correlation between economic conditions and inequity in unmet healthcare need. Nonetheless, this effect disappeared when controlling for other strength indictors of PC in the multiple regression models.

To the best of our knowledge, this is the first study to describe an association between the strength of PC systems and inequity in unmet healthcare need at the macro level; however the authors stress the explorative nature of this study. Given the impact of context on the perception of unmet need, we invite further research exploring this association at the micro level (i.e., explaining unmet need at the individual level by means of strength of the relevant PC system for this individual).

### Strengths and limitations

The operationalisation of unmet need in this study, consistent with the definition of Carr and Wolfe [22], has two limitations. According to this definition, only objective clinically-assessed needs that are not satisfied by appropriate healthcare can be considered unmet. Therefore, this definition has the purpose of detecting subjective or self-assessed unmet health expectations, which are not always clinically grounded. Subjective interpretation of unmet healthcare need is also highly dependent on patient context. Country-specific social and cultural factors (e.g., patient expectations) can influence the evaluation of unmet need [21]. Secondly, the definition of Carr and Wolfe [22] neglects unperceived (but objectively clinically grounded) unmet healthcare need [24]. Notwithstanding these two limitations, this definition is the most suitable method for measuring unmet healthcare need. This subjective assessment of unmet healthcare need perceives the patient to be the best assessor of their health status and of whether they have received the most convenient healthcare [23]. Because the question used in EU-SILC not only probes unmet medical healthcare need but also dental need (which is not relevant to this study), it overestimates the prevalence of unmet need. Also, due to lack of recent data for all included countries, data for different time-periods are used, which could influence the study results. Finally, this study is limited to 31 countries, which, from a statistical point of view, is not optimal [21]. Nevertheless, with this paper, we aimed to take an important step forward in understanding the association between the strength of PC and inequity in unmet healthcare need.

### Policy recommendations

From a policy perspective, our results suggest that policymakers should focus on making PC more accessible and expanding the PC workforce in order to reduce the inequity in unmet healthcare need. Policymakers are therefore urged to develop multidimensional and differentiated legislation that will reduce barriers to care access [23]. In order for enhanced accessibility, we recognise the importance of universal health coverage [52]. As mentioned previously, the US took an important step forward in 2010 with the implementation of the Affordable Care Act. However, the significant association we found between inequity and the Gini coefficient for income inequality shows that in order to reduce inequity, policymakers should first attempt to eliminate income inequality. Only then can strengthening PC systems (i.e., increasing the accessibility of PC and developing the PC workforce) influence inequity in unmet healthcare need. Note that the purpose of this study is to explain the association between the strength of PC systems and inequity in unmet need at the macro level rather than the association between the strength of PC systems and the prevalence of unmet need. The latter association requires further investigation.
